# Research on cross regional emergency material scheduling algorithm based on seed optimization algorithm

**DOI:** 10.1038/s41598-023-47538-2

**Published:** 2023-12-11

**Authors:** Jinbao Li, Lichong Cui, Huayu Chu, Lei Su, Junsheng Wang

**Affiliations:** State Grid Hebei Procurement Company, Shijiazhuang, 050000 Hebei China

**Keywords:** Engineering, Mathematics and computing

## Abstract

In order to improve the response capability of cross regional emergency material scheduling (CREMS), a CREMS algorithm based on seed optimization algorithm is proposed. Construct a segmented regional grid distribution model structure for CREMS, use a grid matching algorithm based on block link distribution to construct the optimization objective function during the emergency material scheduling process, use variable neighborhood search technology to solve the diversity problem of cluster optimization in CREMS, and combine seed optimization algorithms for combination control and recursive analysis in the emergency material scheduling process. Based on the combination of deep learning and reinforcement learning, the optimal route and configuration scheme design for CREMS process is achieved. The simulation results show that this method has better active configuration capability, better path optimization capability and stronger spatial regional planning capability for CREMS.

## Introduction

Natural disasters are one of the important reasons for the increased demand for emergency supplies across regions. According to historical data, natural disasters such as earthquakes, hurricanes, and floods occur on a global scale. For example, according to data from the International Federation of Red Cross/Red Crescent Societies, the 2010 Haiti earthquake resulted in over 220,000 deaths and over 2 million people losing their homes. In 2011, a 9.0 magnitude earthquake and tsunami triggered a tsunami in northeastern Japan, resulting in over 15,000 deaths and 270,000 homelessness. In 2017, Hurricane Harvey hit Texas, causing 82 deaths and an estimated $125 billion in economic losses. Indonesia suffered severe floods in 2020, resulting in approximately 400 deaths and 100,000 forced to leave their homes^1^.

With the continuous improvement of national emergency response capabilities, research on the timeliness and optimization of emergency material scheduling has received attention. In the event of sudden public safety incidents and natural disasters, it is necessary to achieve cross regional and block scheduling of emergency materials. By constructing an emergency logistics support system, a cross regional guarantee and path planning model for emergency material scheduling is established to improve the timeliness of scheduling in emergency logistics. By studying the timeliness of emergency logistics and analyzing the characteristics of major public health emergencies, a new scheduling and distribution system is constructed under important links such as material scheduling. Research on CREMS algorithms, by developing reasonable and efficient vehicle driving routes, optimize the path of rescue materials (such as food, drugs, medical equipment, clothing, etc.) from different aspects of material emergency scheduling work and consider cross regional scheduling^2^. Transport from material distribution centers to various disaster stricken points in the disaster area, improve the emergency management ability and response level of material emergency scheduling. The research on related material scheduling algorithms has received great attention.

At present, the increase in global customer demand and emergency events has led to an increasing demand for accurate prediction and scheduling of emergency supplies. The importance of emergency material scheduling algorithms for accurately predicting and scheduling different customer needs, optimizing resource allocation, and emergency response is becoming increasingly prominent^3^. By applying algorithms, the ability to seamlessly allocate emergency supplies can be effectively improved, meeting cross regional customer needs, and reducing costs and time pressure in emergency situations. In addition, algorithms can also assist institutions in cross departmental cooperation, coordinate material scheduling and resource management, and improve the efficiency of emergency response and disaster management^4^. According to the latest trend analysis, emergency material scheduling algorithms will continue to be widely researched and applied, providing more effective support for emergency response and disaster management.

In response to the problems existing in traditional methods, this paper proposes a CREMS algorithm based on seed optimization algorithm. Firstly, a block regional grid distribution model structure for CREMS is constructed, and the multi-objective adaptive particle swarm optimization and seed swarm optimization models for emergency material scheduling are constructed. Then, the grid matching algorithm of block link distribution is used to construct the optimization objective function in the process of emergency material scheduling. Combined with variable neighborhood search technology, the cluster optimization diversity problem in CREMS is solved, The seed optimization algorithm has been established, and the combination control and recursive analysis in the emergency material scheduling process have been combined. Based on the combination of deep learning and reinforcement learning, the optimal route and configuration scheme design for CREMS process has been achieved. Finally, experimental testing and simulation analysis have been used to demonstrate the superiority of this method in improving the ability of CREMS.

## Literature review

In the study of CREMS, due to the different specific situations of disasters, the objectives and influencing factors considered in CREMS also have certain differences. In order to meet the special needs of different disaster situations, the quality of scheduling schemes depends on the optimization ability of problem solving algorithms, such as in achieving single objective problems.

Zhang^5^ has achieved significant results in CREMS problems through the use of Lagrange relaxation and linear programming models, as well as multiple ant colony optimization algorithms and mixed integer programming models. These studies provide effective methods and tools for emergency material scheduling to reduce transportation costs, improve food distribution efficiency, and ensure emergency scheduling safety. Dan^6^ explored different objective criteria such as transaction time, cost, security, and fairness, and adopted an improved particle swarm optimization algorithm to solve the emergency material scheduling model. This approach can make the distribution of emergency supplies more fair and feasible, ensuring the maximization of benefits. In filling the research gaps in the literature, these studies provide specific solutions and methods for CREMS. They comprehensively consider problems from multiple perspectives and objective standards, and provide theoretical and practical contributions to the challenges in this field. This helps to improve the existing research framework, provide more effective scheduling algorithms and decision support to meet the actual needs of material scheduling in emergency response. Ransikarbum et al.^7^ is based on goal programming for post disaster decision-making, used for comprehensive relief allocation and early network recovery. This method focuses on solving post disaster relief allocation and network recovery problems by developing a goal planning model. The specific implementation process requires investigating the existing disaster relief situation and network recovery situation, then establishing a mathematical model, defining the objective function and constraint conditions, and using appropriate algorithms to solve the model to obtain the optimal comprehensive decision-making plan. These methods can effectively solve the single objective emergency material scheduling problem, reduce transportation costs, and improve food distribution efficiency. This method has strong optimization ability for emergency material scheduling and can comprehensively consider various factors, such as transportation time, cost, safety, etc. However, for complex multi-objective emergency material scheduling problems, this method may not be able to obtain the optimal solution and needs to be combined with other methods to solve.

Ransikarbum et al.^8^ used a hybrid NSGA-II algorithm for multi-objective optimization of post disaster relief allocation and short-term network recovery. This method uses a hybrid NSGA-II algorithm to solve the problems of post disaster relief allocation and network recovery, while considering multiple objectives simultaneously. By balancing the relationships between different objectives, the optimal comprehensive decision-making solution is obtained. The specific implementation process includes using the hybrid NSGA-II algorithm to model the problem, setting appropriate objective functions and constraints, adjusting parameters, and running the algorithm to obtain optimization results, in order to achieve dual objective optimization. These research methods provide effective methods for understanding post disaster decision-making, disaster relief allocation, and network recovery by considering multiple factors and objectives. They help fill some research gaps in literature on comprehensive disaster decision-making and multi-objective optimization problems, and provide useful reference and guidance for decision-makers in actual emergency situations. Zhang^9^ utilized ArcGIS software for network analysis and other tools, as well as through data preparation, data processing, and network analysis processes, to study the scheduling of emergency supplies, which has feasibility and convenience in solving scheduling problems. Jiang^10^ proposes a dynamic attention model based on an improved gated recurrent unit. A dynamic encoder framework is used to track changes in node demand and update node information. The improved gated recurrent unit is embedded between encoders to enhance the model's representation capability. By weighting the node information from the previous time, current time, and initial time, a more representative node embedding is obtained, thereby minimizing the cost of material distribution. These methods can be used to solve the problem of emergency material scheduling, which is feasible and convenient. Simulate and optimize emergency material scheduling using network analysis and data processing tools. However, for complex emergency material scheduling problems, this method may not be able to obtain the optimal solution and needs to be combined with other methods to solve.

Mirzaei-Nasirabad^11^ proposes a novel multi-objective mathematical model to solve the problem of dynamically allocating trucks, with the aim of minimizing fleet waiting times and deviations from the path production requirements established by the allocation planning. To evaluate the performance of the proposed model, three different heuristic methods are developed, including minimizing shovel idle time, minimizing ratio variance, and minimizing the deviation from the allocation planning. Yingying^12^ an improved K-means algorithm is used to determine truck docking points, and a genetic simulated annealing algorithm is employed to optimize the joint delivery routes of trucks and drones. This approach effectively reduces the total operating costs. These methods can solve multi-objective logistics scheduling problems and obtain the optimal comprehensive decision-making solution. However, the problem lies in the high complexity of the algorithm, which not only requires a high computational environment, but also easily falls into local optima.

The potential drawbacks of these methods include complex algorithms, requiring a large amount of data, model assumptions, local optimal solution problems, and parameter settings, which require further improvement to improve accuracy, applicability, and interpretability.

The method in this article is based on seed optimization algorithm, and there are some differences in application methods, case studies, variables, and disaster operation stages compared to the aforementioned methods such as Lagrange relaxation, linear programming model, multi ant colony optimization algorithm, and mixed integer programming model. Firstly, the method proposed in this article is based on the seed optimization algorithm, which is a heuristic algorithm that can solve complex optimization problems. This algorithm seeks the optimal solution by simulating the propagation and crossover of seeds in different solution spaces. Compared to other algorithms, seed optimization algorithms have strong global search ability and adaptability. Secondly, this study employed different case studies and variables. Specifically, the research object is the cross regional emergency material scheduling problem. In terms of case studies, this article may have selected different types of natural disasters or emergencies, and determined corresponding material needs and scheduling goals for each situation. In terms of variables, this article may consider factors such as material supply and demand relationship, material distribution routes, transportation costs, etc. In addition, this article may also focus on different aspects of the disaster operation phase. This may include stages such as resource preparation and reserve during the pre disaster period, material scheduling and distribution after a disaster, and post disaster recovery and reconstruction. By considering and optimizing scheduling problems at different stages, the efficiency and flexibility of rescue operations can be improved.

Overall, the method proposed in this article is based on seed optimization algorithms, which differ from the previous methods in terms of application methods, case studies, variables, and disaster operation stages. This method helps to solve the problem of cross regional emergency material scheduling and provides a feasible and convenient application method.

## Parameters/variables and definitions

The relevant parameters in the article are defined in Table 1.

**Table 1:** Parameter interpretation.

Parameter	Explain
i	The nodes
j	The nodes
P	The transport cost
t	The transport moment
β	Assigns the pheromone evolution generations for transport
s	The vector of the fuzzy distribution
c	The fitness value
u0	The individual divergence
H	The pathway network structure
$\phi$	The diversification selection weights
$\lambda$	The importance of transporting materials
h	The single run of the vehicle passing through the node
D	The maximum
$\tau_{ij} \left( t \right)$	The pheromone feature distribution vector set of cross regional emergency material dispatching route search, which is based on the shortest path variation
$\eta_{ij} (t)$	The energy consumed by the pheromone dispersion
α	The pheromone capacity, and s indicates the dispersion of the pheromones
$\{ x_{k - 1}^{i} ,w_{k - 1}^{i} \}$	The fusion parameter distribution set of cross regional material dispatching within the k-th perception range.p is the prior probability
$p(x_{0} )$	The estimated prior probability of cross regional emergency material dispatching vehicles
rs	The spatial distributed random degree
x	The H, O, C, S, L static attribute mapping value
K	The degree of discrimination
$v_{id}^{t}$	The optimal eigensolutions of the i mixed quantum genetic individual at S and t time
SD	The statistical classification of pheromone for cross regional emergency material dispatching vehicle optimal dispatching
t	The number of nodes for cross regional emergency material dispatching vehicle optimal dispatching
$c_{3} \cdot rand\left( {} \right)$,$c_{4} \cdot rand\left( {} \right)$	Random distribution operators for CREMS route planning
$x_{ij} \in \left[ {x_{\min } ,x_{\max } } \right]$,$\tilde{X}_{i} = (\tilde{x}_{i1} ,\tilde{x}_{i2} , \ldots ,\tilde{x}_{ij} ,\tilde{x}_{iD} )$	The spatial information gain of CREMS route planning
$e_{p}$	The weight deviation of each node in the depot
$e_{g}$	The total distance traveled
$e_{0p}$	The amount of pheromone rules to be sown
$e_{0g}$	The threshold value of the deviation between the current value and the current global optimal value

## Assumptions

In the proposed CREMS algorithm based on seed optimization algorithm, the main assumption is that the scheduling of emergency supplies is feasible, that is, within a given time and resource limit, the required emergency supplies can be successfully transported from the supply point to the demand point. This assumption is based on an understanding of the transportation network and traffic conditions, as well as predictions of the demand and supply of emergency supplies. It is also assumed that the supply and demand of emergency supplies are known and can accurately predict future demand. This assumption is crucial for the accuracy of the model and algorithm. Another assumption is that the transportation time and cost can be accurately estimated during the emergency material scheduling process. This assumption is crucial for the efficiency of the model and algorithm. In addition, it is also assumed that there will be no shortage of supply during the scheduling process of emergency supplies. It is also assumed that during the emergency material dispatch process, all transportation is reliable, that is, there will be no unexpected situations during the transportation process.

## A grid distribution model and feature analysis for material scheduling in segmented regions

### Blocked area grid distribution model

**Figure 1:**
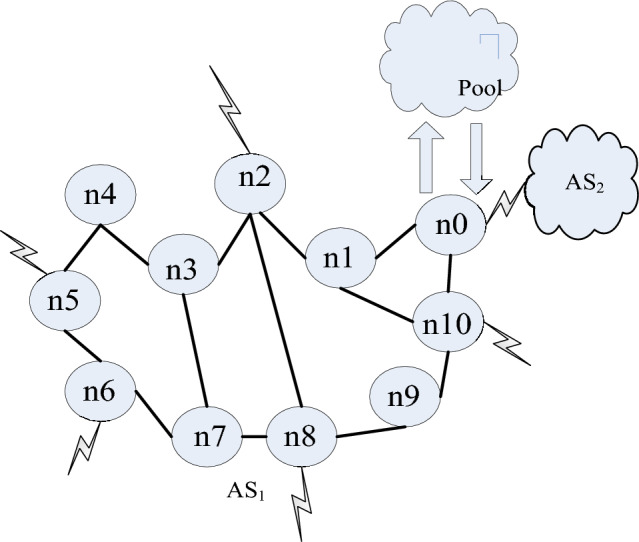
Grid distribution model for segmented regions.

In order to achieve CREMS based on seed optimization algorithm, a segmented regional grid distribution model structure for CREMS is first constructed, and the vehicle scheduling model is analyzed. Based on the total distance traveled by individual W along the route, and according to individual movement rules^13^, as the number of population updates increases, the majority of individuals in the population will quickly move. The rule function is:

1 $$ \begin{aligned} \varphi (i,t) & = \mathop \prod \limits_{j = 1, \ldots ,n} \{ P(j,t - 1)(1 - \beta ) + [1 - P(j,t - 1)]\} \\ & \quad = \mathop \prod \limits_{j = 1, \ldots ,n} [1 - \beta P(j,t - 1)] \\ \end{aligned} $$

where *i*, *j* are the nodes, *P* is the transport cost, *t* is the transport moment, and *β* assigns the pheromone evolution generations for transport. Since the number of adjacent nodes j of node i is n, cross regional emergency material dispatching route planning and adaptive control are carried out under the pheromone guidance rule of cross regional emergency material dispatching route distribution^14^. The optimization problem of individuals searching for the best individuals in the region with large solution space is combined with individual screening to form a new generation of population correlation detection method, and the evolution algebra of cross regional emergency material dispatching route distribution is obtained. In the D-dimensional space, the fuzzy distribution vector fusion parameters for CREMS are:

2 $$ S = (s_{ij} )_{(n + 1) \times (n + 1)} ,\quad (i,j = 0,1, \ldots ,n) $$

where *s* is the vector of the fuzzy distribution. The optimal solution of the problem on a global scale is obtained, randomly select a random number $0 \le q \le 1$, and if $q \ge q_{0}$, perform position analysis and node control of the CREMS route^15^. Obtain a node distribution position of $X_{{{\text{best}}}}$, while maintaining individual differences. If only the fitness value is used to screen individuals, automatically adjust the cross regional emergency material dispatching route, build an intelligent cross regional emergency material dispatching route network structure model^16^, and obtain the path selection rules of population diversity awareness as follows:

3 $$ \begin{aligned} \inf \{ E[c_{1} ,c_{2} ,\phi |u_{0} ]\} & = \lambda_{1} \int_{\Omega } {(u_{0} - c_{1} )^{2} } H\left( \phi \right)dxdy \\ & \quad + \lambda_{2} \int_{\Omega } {(u_{0} - c_{2} )^{2} } \left( {1 - H\left( \phi \right)} \right)dxdy \\ \end{aligned} $$

where c represents the fitness value, u_0_ represents the individual divergence, and H represents the pathway network structure, $\phi$ represents the diversification selection weights, $\lambda$ represents the importance of transporting materials. In different regions of the solution space, analyze individual structural models and represent the clustering center of the intelligent CREMS route with a quaternion G^17^, denoted as $c$. There are m intelligent CREMS route nodes, denoted as $A_{1}$, $A_{2}$…$A_{n}$, and the departure time of vehicles at each key point is denoted as $a_{1}$, $a_{2}$…$a_{n}$. The key point j is used as the location of vehicle k. The grid distribution mode of the segmented area is shown in Fig. 1.

Based on the actual situation of the MED-DRC problem, the scheduling nodes are divided into N clusters to ensure that there is only one vehicle entering and exiting each affected point^18^. The number of times the comprehensive information is transmitted is:

4 $$ \left( {1 + \sum\limits_{h = 2}^{{\frac{D + 1}{2}}} {8(h - 1) \cdot h} } \right) \cdot \lambda a^{2} = \left( {\frac{{D^{3} - D}}{3} + D^{2} } \right) \cdot \lambda a^{2} $$

where h is the single run of the vehicle passing through the node, and the maximum is D. The total load of each vehicle departing from the distribution center is $N$, and the time cost spent by the vehicle during driving is composed of:

5 $$ \left( {\frac{{D^{3} - D}}{3} + D^{2} } \right) \cdot \lambda a^{2} \cdot \frac{N}{{\lambda D^{2} a^{2} }} = \left( {\frac{D}{3} - \frac{1}{3D} + 1} \right) \cdot N $$

At the initial moment, it is necessary to obtain the total number of cross regional emergency material dispatch routes as $M$ based on the current known location^19^, demand, and road information of the affected point. The angle between the affected point and the origin line is:

6 $$ \begin{aligned} T_{{{\text{inter}}}} & = \left( {\frac{N}{{\lambda D^{2} a^{2} }} - 1} \right) \cdot D \cdot M + \frac{D}{2} \cdot M \\ & \quad = \frac{NM}{{\lambda a^{2} }} \cdot \frac{1}{D} - \frac{M}{2} \cdot D \\ \end{aligned} $$

wherein the value of D has an autocorrelation matching relationship with AS. When AS is less than, the angles of each affected point in polar coordinates are calculated^20^, from the origin to all affected points, to obtain:

7 $$ D^{ * } = \left\{ {\begin{array}{*{20}c} {\sqrt {\frac{{\frac{3M}{{\lambda a^{2} }} - 1}}{{1 - \frac{3M}{{2N}}}}} ,M < \frac{2}{9}N;} \\ {D_{MAX} ,\frac{2}{9}N \le M \le N.} \\ \end{array} } \right. $$

Starting from the polar axis, two adjacent disaster points and the origin are sequentially calculated to form a single cluster^21^. The disaster point with the largest angle with the polar axis is selected as the starting point for counterclockwise scanning, and information extraction and clustering processing are carried out.

### Emergency material dispatch target model

Add the affected points to the planned set of affected points, generate emergency material scheduling based on the shortest path mutation method, construct a hybrid quantum genetic model^22^, and obtain the dynamic model parameter distribution set for cross regional material scheduling as follows:

8 $$ p_{ij}^{k} \left( t \right) = \left\{ {\begin{array}{*{20}c} {\frac{{\left[ {\tau_{ij} \left( t \right)} \right]^{\alpha } \left[ {\eta_{ij} (t)} \right]^{\beta } }}{{\sum\limits_{{s \in allowed_{k} }} {\left[ {\tau_{is} \left( t \right)} \right]^{\alpha } \left[ {\eta_{is} (t)} \right]^{\beta } } }},} & {if{\kern 1pt} j \in allowed_{k} } \\ {0,} & {else} \\ \end{array} } \right. $$

wherein $\tau_{ij} \left( t \right)$ refers to the pheromone feature distribution vector set of cross regional emergency material dispatching route search, which is based on the shortest path variation, $\eta_{ij} (t)$ represents the energy consumed by the pheromone dispersion, α represents the pheromone capacity, and s indicates the dispersion of the pheromones^23^. $\{ x_{k - 1}^{i} ,w_{k - 1}^{i} \}$ refers to the fusion parameter distribution set of cross regional material dispatching within the k-th perception range.

Suppose that the pheromone strong degree distribution set of time cross region material dispatching is z_k_. Hierarchical clustering is used to divide the individuals in the current population equally according to their differences^24^, and the fuzzy weight value of the optimal dispatching of emergency dispatching vehicles is obtained:

9 $$ \tilde{w}_{k}^{i} = \tilde{w}_{k - 1}^{i} \frac{{p(z_{k}^{{}} /\tilde{x}_{k}^{i} )p(\tilde{x}_{k}^{i} /x_{k - 1}^{i} )}}{{q(\tilde{x}_{k}^{i} /x_{k - 1}^{i} )}} $$

where p is the prior probability. Initialize the path distribution of cross regional emergency material dispatching vehicles, give the estimated prior probability $p(x_{0} )$ of cross regional emergency material dispatching vehicles^25^, and record the individuals with the best quality in each group as $\{ x_{0}^{i} ,i = 1,2, \ldots  {\text{N} }\}$. Using seed swarm algorithm for CREMS vehicle optimization scheduling and path planning^26^, initialize the adaptive weight to 1/N, and normalize the weight of the path space planning for CREMS vehicle optimization scheduling:

10 $$ \tilde{\tilde{w}}_{k}^{i} = \tilde{w}_{k}^{i} /\sum\limits_{i = 1}^{N} {w_{k}^{i} } $$

The mathematical model for optimizing the scheduling of cross regional emergency material dispatch vehicles is constructed, determining the path at the current point and moving target^27^. Combining with the spatial distribution intensity of cross regional emergency material dispatch vehicles, the difference goal planning parameter between individuals i and j is obtained as $N_{eff} \approx 1/\sum\limits_{i = 1}^{N} {(\tilde{\tilde{w}}_{k}^{i} )^{2} }$. The multi target Pareto mapping method is used to design the path planning for cross regional emergency material dispatch^28^. The node space distribution set for CREMS vehicle scheduling is obtained as $\{ W_{final} \} = \{ \{ W_{H} \} ,\{ W_{C} \} ,\{ W_{O} \} \}$, and the population position of the target point is found. The fuzzy constraint state parameters for CREMS vehicle scheduling are estimated as:

11 $$ \hat{x}_{k}^{{}} = \sum\limits_{i = 1}^{N} {w_{k}^{i} } x_{k}^{i} $$

In the initial stage of operation, the positions of quantum group individuals for CREMS vehicle scheduling at time $k + 1$ are calculated, and the iterative formula for path fusion is obtained:

12 $$ x_{i} (k + 1) = x_{i} (k) + s\left( {\frac{{x_{j} (k) - x_{i} (k)}}{{\left\| {x_{j} (k) - x_{i} (k)} \right\|}}} \right) $$

where $\left\| {\vec{x}} \right\|$ represents the Euler function of $\vec{x}$. The spatial distribution weight coefficient of vehicle scheduling for cross regional emergency material dispatch is:

13 $$ r_{d}^{i} (k + 1) = \min \{ r_{S}^{{}} ,\max \{ 0,r_{d}^{i} (k) + \beta (n_{i} - \left| {N_{i} (k)} \right|)\} \} $$

where the *r*_*s*_ are the spatial distributed random degree. Based on the above analysis, identify the individual diversity with the best quality in the current population based on the differences and similarities between the two^29^.

## Optimization of emergency material scheduling algorithm

### Seed swarm learning algorithm for cross regional scheduling of emergency supplies

Using multi-objective Pareto mapping method for path planning design of CREMS, analyzing the same path segments in individual i and guiding individual j. Using static attribute fusion method, the estimated target state parameters for CREMS vehicle optimization scheduling are:

14 $$ \begin{aligned} x_{F}^{i} & = \frac{1}{N}\{ \sum\limits_{i = 1}^{m} {x_{H}^{i} + } \sum\limits_{i = 1}^{N - m - a} {x_{O}^{i} + } \sum\limits_{i = 1}^{a} {x_{C}^{i} } \} = \frac{1}{N}\sum\limits_{i = 1}^{m + a} {x_{H}^{i} + } \frac{1}{N}\sum\limits_{i = 1}^{N - m - a} {x_{O}^{i} } \\ & \quad = \frac{1}{N}\sum\limits_{i = 1}^{m + a} {x_{H}^{i} + } \frac{1}{N}\sum\limits_{i = 1}^{N - m - a} {x_{S}^{i} (1 - Kd_{i}^{\max } ) + } \frac{1}{N}\sum\limits_{i = 1}^{N - m - a} {Kd_{i}^{\max } x_{L}^{i} } \\ \end{aligned} $$

where x represents the H, O, C, S, L static attribute mapping value, and K represents the degree of discrimination. According to the above settings, guide the individual oriented local small range search, and combine the reference factors of environment, map, and pheromone^30^, initialize N hybrid genetic populations for optimal scheduling of cross regional emergency materials dispatching vehicles. The movable spatial distribution model of all individuals is $\left( {X_{1} \left( 0 \right),X_{2} \left( 0 \right), \, \ldots ,X_{N} \left( 0 \right)} \right)$, so as to update the path optimal scheduling of cross regional emergency materials dispatching, obtain an instance initialization parameter distribution model for cross regional emergency material dispatch routes, optimize the spatial configuration parameters of cross regional emergency material dispatch vehicles, and when the number of paths is equal to m, obtain the inertia distribution weight for optimizing cross regional emergency material dispatch routes:

15 $$ \begin{aligned} & V_{t + 1,i} = \underbrace {{\omega \times V_{t,i} }}_{(mementum)} + C_{1} \underbrace {{ \times rand() \times (p_{t,i} - X_{t,i} )}}_{(Cognitive\;Component)} \\ & \quad \underbrace {{C_{2} \times rand() \times (p_{gt} - X_{t,i} )}}_{(Social\,Component)} \\ \end{aligned} $$

wherein ${\mathbf{X}}_{t + 1,i} = {\mathbf{X}}_{t,i} + {\mathbf{V}}_{t + 1,i}$. The background monitoring thread is established, and the initial CREMS and scheduling thread parameters are set to $tadu_{k}$ to establish a population array fusion parameter set for CREMS and vehicle optimization scheduling. Conduct global optimal path control for CREMS and scheduling, set the mixed quantum group distribution of individual optimal position *p*_*i*_ and global optimal parameter set pg for CREMS vehicle optimization scheduling, solve the statistical characteristic quantity S for CREMS vehicle optimization scheduling, and combine the information concentration of CREMS vehicle optimization scheduling within the range of CREMS vehicle movement rules, The main behavior and decision-making modeling parameters of cross regional emergency material dispatch vehicles are represented as:

16 $$ \tau_{ij} = \frac{{k_{ij} }}{m} $$

By performing feature separation and information reconstruction on the above equation, the optimal individual location for cross regional emergency material dispatch vehicle optimization scheduling is obtained as follows:

17 $$ v_{id}^{t} = v_{id}^{t - 1} + (x_{id}^{t} - x_{d}^{*} ).f_{i} $$

In the formula, $v_{id}^{t}$ and $v_{id}^{t - 1}$ are the optimal eigensolutions of the first mixed quantum genetic individual at S and $t - 1$ time respectively, SD represents the statistical classification of pheromone for cross regional emergency material dispatching vehicle optimal dispatching, $t - 1$ represents the number of nodes for cross regional emergency material dispatching vehicle optimal dispatching, it constructs the hybrid quantum genetic evolution optimization model for cross regional emergency material dispatching vehicle optimal dispatching.

### Optimization and implementation of emergency material dispatch model

**Figure 2:**
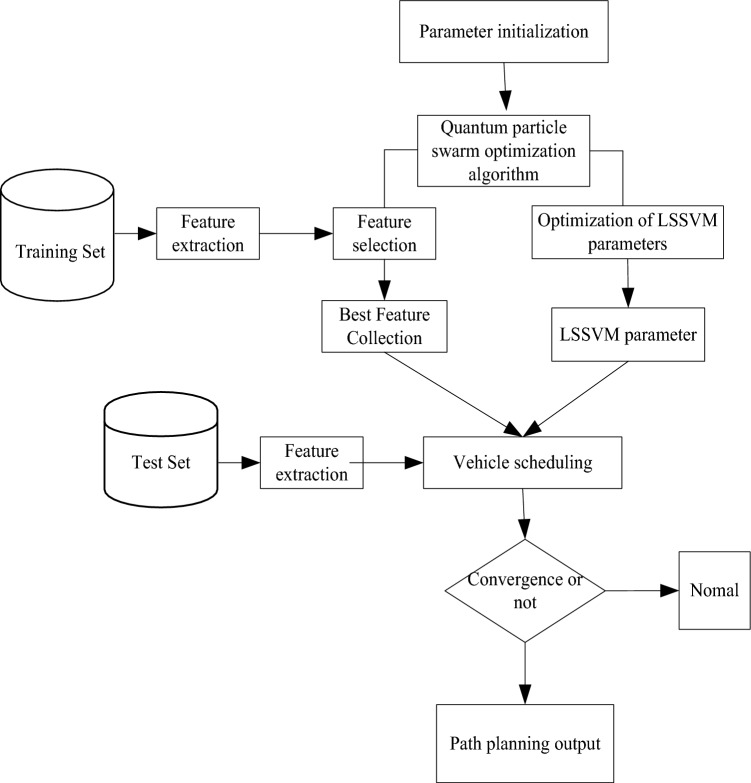
Implementation process of optimizing vehicle scheduling for cross regional emergency material dispatch.

Each individual in the population can reflect the vehicles required for material distribution to all affected points. The distance traveled by vehicles to visit a certain affected point is related to the relative position of the affected point in the vehicle depot. The iterative equation for global extremum or individual extremum seeking in the path planning of CREMS is as follows:

18 $$ \begin{aligned} v_{i,d}^{k + 1} & = \omega \cdot v_{i,d}^{k} + c_{1} \cdot rand\left( {} \right) \cdot \\ & \quad \left( {c_{3} \cdot rand\left( {} \right) \cdot pbest_{i,d}^{k} - x_{i,d}^{k} } \right) \\ & \quad + c_{2} \cdot rand\left( {} \right) \cdot \left( {c_{4} \cdot rand\left( {} \right) \cdot gbest_{d}^{k} - x_{i,d}^{k} } \right) \\ \end{aligned} $$

wherein $c_{3} \cdot rand\left( {} \right)$ and $c_{4} \cdot rand\left( {} \right)$ are random distribution operators for CREMS route planning. The expression for calculating the selected vehicle depot LC, except for the distribution center node, is:

19 $$ c_{3} \cdot rand\left( {} \right) = \left\{ {\begin{array}{*{20}l} {1,} \hfill & {e_{p} > e_{0p} } \hfill \\ {c_{3} \cdot rand\left( {} \right),} \hfill & {e_{p} \le e_{0p} } \hfill \\ \end{array} } \right. $$

20 $$ c_{4} \cdot rand\left( {} \right) = \left\{ {\begin{array}{*{20}l} {1,} \hfill & {e_{g} > e_{0g} } \hfill \\ {c_{4} \cdot rand\left( {} \right),} \hfill & {e_{g} \le e_{0g} } \hfill \\ \end{array} } \right. $$

wherein $x_{ij} \in \left[ {x_{\min } ,x_{\max } } \right]$ and $\tilde{X}_{i} = (\tilde{x}_{i1} ,\tilde{x}_{i2} , \ldots ,\tilde{x}_{ij} ,\tilde{x}_{iD} )$ are referred to as the spatial information gain of CREMS route planning, $\tilde{x}_{ij} = x_{\max } + x_{\min } - x_{ij}$ represents the configuration parameters for seed group evolutionary optimization, and $c_{4}$ represents the spatial search range of CREMS route; $e_{p}$ represents the weight deviation of each node in the depot, and $e_{g}$ represents the total distance traveled; $e_{0p}$ represents the amount of pheromone rules to be sown, and $e_{0g}$ represents the threshold value of the deviation between the current value and the current global optimal value. According to each group of disaster affected points, an artificial intelligence algorithm is developed based on improved scanning algorithms to optimize the scheduling of cross regional emergency material dispatch vehicles, achieving cross regional emergency material dispatch vehicle optimization scheduling and artificial intelligence control. Based on the above analysis, cross regional emergency material dispatch vehicle optimization scheduling and artificial intelligence control are achieved. The implementation process is shown in Fig. 2.

## Simulation testing

**Figure 3:**
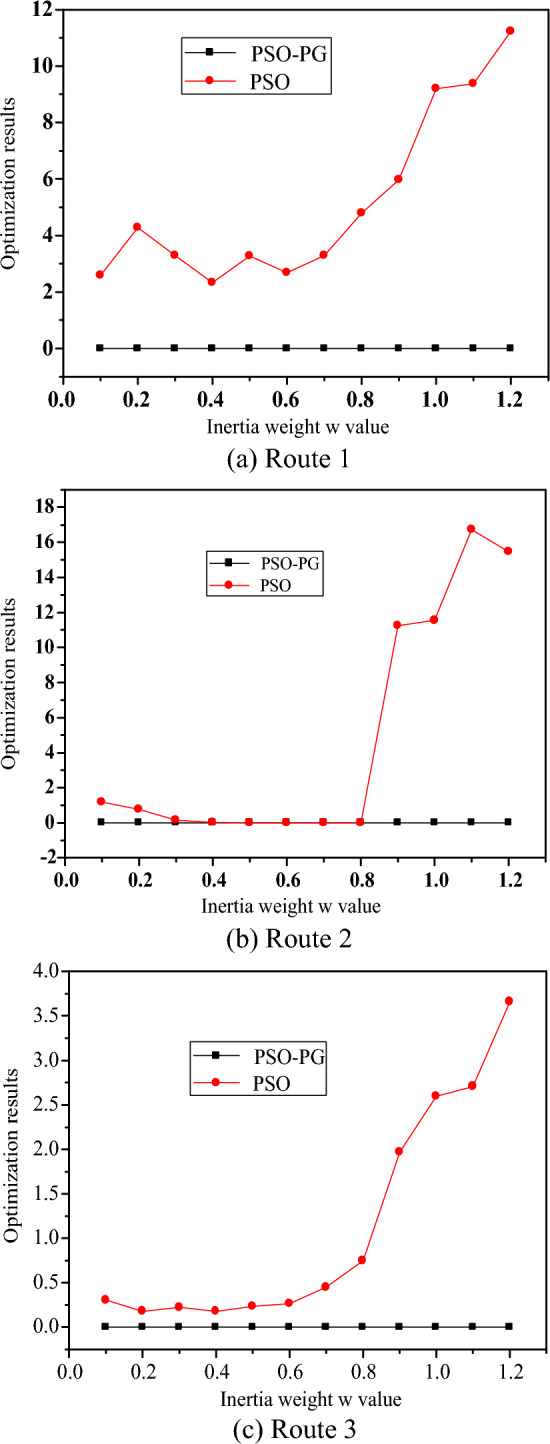
Convergence value output of vehicle path planning.

In order to verify the application performance of the method proposed in this article in achieving cross regional emergency material dispatch vehicle optimization scheduling, simulation tests were conducted. Computers using Intel Core i7 processors are equipped with Windows 10 operating system and SSD solid-state drives as storage devices, with a memory configuration of 16 GB. Using Java as the programming language for this experiment. The scale of the vehicles for cross regional emergency material dispatch was 300, and the model parameters were optimized based on different node numbers. The global optimal value of vehicle path distribution was $min\left( {f_{{6}} } \right) = f_{{6}} \left( {0,0, \ldots ,0} \right) = 0$. A dynamic attention model without IGRU was used for dynamic optimization, and the fuzzy matching parameter was $c_{1ini} = {3}$, and the adjacent feature matching coefficient was 1.35. When the minimum iterative parameter of quantum group optimization is $c_{1fin} = 0.{35}$, and the number of nodes is 10, 20, 50, and 100 respectively, the same hyperparameter is used to train the DAM model, and the iterative step size of seed group evolution is $c_{2fin} = {5}.{4}$, and the maximum detection threshold is $c_{2fin} = {5}.{4}$. Each test set contains 2000 samples, and the minimum detection threshold is. According to the above parameter settings, each $\mu_{{{\text{min}}}} = 0.{23}$ conducts 30 repeated experiments and records the average value as the final result. The convergence value output of the current cross regional emergency material dispatch vehicle optimal path planning is shown in the Fig. 3. Where, PSO-PG is Particle Swarm Optimization-Particle Swarm Optimization, PSO is Particle Swarm Optimization.

**Figure 4:**
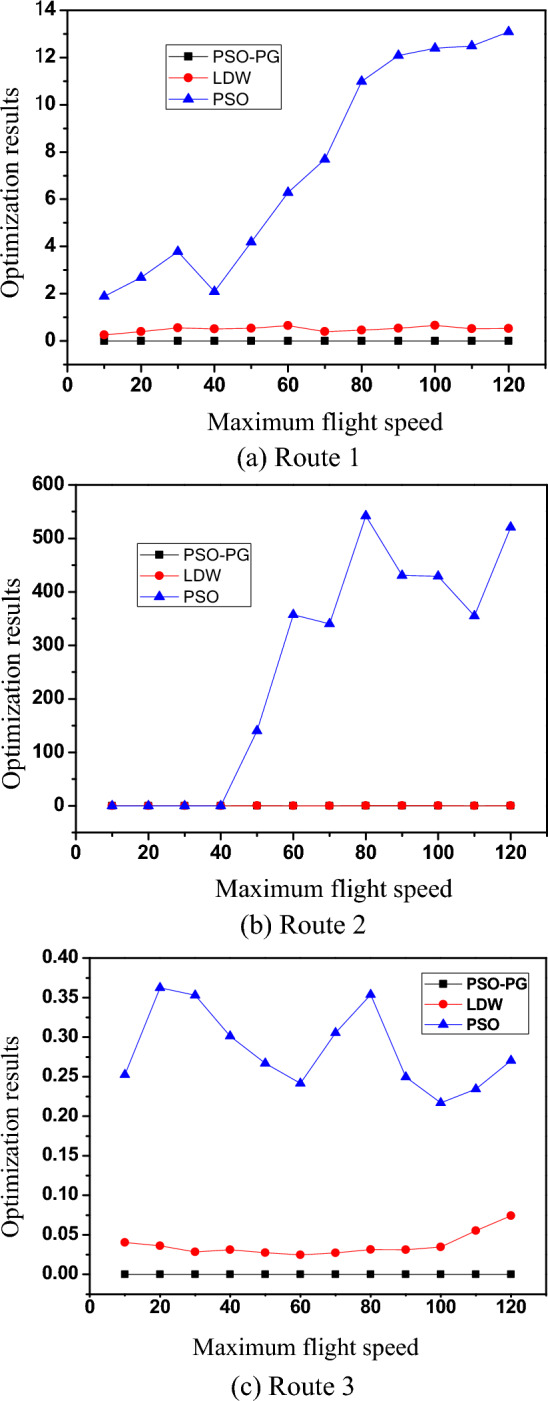
Optimal solution under different quantum population sizes.

By analyzing Fig. 3, it can be concluded that the method proposed in this paper can effectively achieve path planning for CREMS vehicles, and test the optimal solutions under different quantum population sizes. The results are shown in Fig. 4. Where, LDW is Learning with Delayed Rewards.

**Figure 5:**
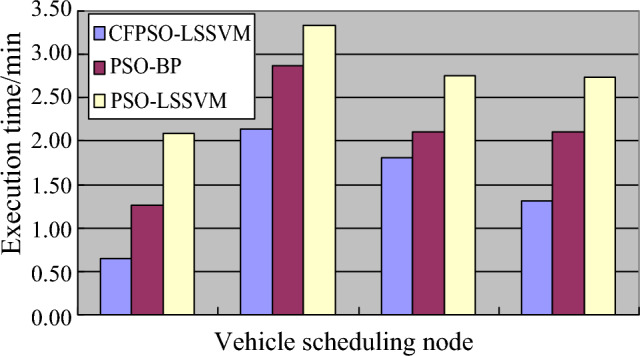
Execution time of vehicle scheduling.

By analyzing Fig. 4, it can be concluded that the optimal solution parameter analysis results for cross regional emergency material dispatch vehicle scheduling and planning using this method are accurate and reliable, improving the dynamic management ability of emergency material dispatch. The execution time of vehicle scheduling was tested and the comparison results are shown in Fig. 5.

Based on the analysis of the above simulation results, it can be concluded that the method proposed in this paper has good optimization ability and strong path planning ability for CREMS vehicles. However, the PSO algorithm and LDW algorithm are almost unable to jump out of local optima, while the PSO-PG algorithm has obtained global optima under different population sizes, indicating that the method proposed in this paper can effectively improve vehicle scheduling efficiency.

Based on the analysis of the simulation experimental results above, the following conclusions can be drawn:

The method proposed in this article has shown good application performance in optimizing the scheduling of vehicles for cross regional emergency material dispatch. By optimizing the number of different nodes, the scale of emergency material dispatch vehicles across regions was determined to be 300, and a dynamic attention model was used for dynamic optimization. The results show that the PSO-PG algorithm obtained the global optimal solution, which has better path planning ability compared to the PSO algorithm and LDW algorithm. When tested under different quantum population scales, this method obtained accurate and reliable optimal solution parameter analysis results, effectively improving the dynamic management ability of emergency material scheduling. In addition, the experimental results also show that this method can execute vehicle scheduling in a relatively short time, further proving the improvement of its scheduling efficiency. In summary, the method proposed in this article demonstrates excellent performance in optimizing vehicle scheduling for CREMS, with optimization and path planning capabilities. Compared with PSO algorithm and LDW algorithm, PSO-PG algorithm can better solve the problem of local optimal solution, thereby effectively improving the efficiency of vehicle scheduling.

## Conclusions

The research paper investigates a seed optimization-based algorithm for cross-regional emergency supplies dispatch. It aims to enhance the emergency management capability and response level of material emergency scheduling by developing efficient vehicle driving routes and considering path optimization and cross-regional scheduling factors. The unique contribution of this paper lies in proposing the CREMS algorithm, which is based on a seed optimization algorithm. By combining deep learning and reinforcement learning techniques, it achieves optimal route and configuration design for the CREMS process. Moreover, the optimization of scheduling information concentration for CREMS vehicles enables effective behavior and decision modeling. The findings of the study highlight the accurate and reliable results obtained from the parameter analysis of CREMS vehicle scheduling and planning using the proposed method. Furthermore, it improves the dynamic management ability of emergency material scheduling and reduces scheduling time. While the current research primarily focuses on optimizing a single objective, there is potential for future research to consider multiple objectives, such as minimizing transportation costs and maximizing service coverage. Designing appropriate multi-objective optimization methods can further enhance the effectiveness of cross-regional emergency supplies dispatch. It is important to acknowledge the limitations of the current study. Further investigation should explore the integration of practical constraints, such as road restrictions and traffic congestion. Additionally, conducting real-world application validations will provide valuable insights into the scalability and applicability of the proposed algorithm. In conclusion, the seed optimization-based algorithm presented in this paper contributes to the field of cross-regional emergency supplies dispatch. Its application has significant practical implications for improving emergency management processes. Future research should focus on addressing identified limitations and exploring additional optimization objectives to enhance performance in real-world scenarios.

## Data Availability

All data generated or analyzed during this study are included in this published article.
